# Tracking the evolution of a single choice

**DOI:** 10.7554/eLife.103059

**Published:** 2024-10-21

**Authors:** Bharath Chandra Talluri, Hendrikje Nienborg

**Affiliations:** 1 Laboratory of Sensorimotor Research, National Eye Institute, National Institutes of Health Bethesda United States

**Keywords:** decision making, reaction time, neuropixels, drift-diffusion, parietal cortex, population code, Rhesus macaque

## Abstract

Measuring the activity of hundreds of neurons in macaque brains simultaneously provides further evidence that drift-diffusion dynamics underlie how decisions are made in the brain.

**Related research article** Steinemann NA, Stine GM, Trautmann EM, Zylberberg A, Wolpert DM, Shadlen MN. 2024. Direct observation of the neural computations underlying a single decision. *eLife* **12**:RP90859. doi: 10.7554/eLife.90859.

Imagine observing a flock of birds migrating south in the fall. How does the group collectively make a turn? If you watch a single bird turn, even multiple times, you still may not be able to answer this question. But if you observe many birds at the same time, the solution may become clearer. This is also true for understanding how the brain makes decisions: analyzing the behavior of multiple neurons simultaneously can provide information that is not available from a single neuron.

The underlying neuronal mechanisms behind decision-making are often explained by a mathematical theory known as the drift-diffusion model (Ratcliff, 1978), particularly for tasks involving choosing between two options. The drift component represents the process of moving towards one option based on evidence that accumulates over time (such as momentary pieces of sensory information), while diffusion represents random variability in the evidence received and how it is processed by the brain. Together, drift and diffusion constitute the signal that is thought to influence what decisions individuals make, and how long it takes.

Previous studies tested this model by recording the activity of single neurons in the lateral intraparietal (LIP) area of macaques while they performed a direction discrimination task where they decided which direction a patch of dots on a screen were moving. While the majority of the dots moved randomly, a proportion travelled in the same direction towards a target on the left- or right-hand side of the screen, which the macaques shifted their gaze towards to indicate their decision (Figure 1A). Averaging this neuronal activity across multiple trials revealed a drift-like signal that gradually increased over time to resemble a ramp-like pattern (Figure 1B; Shadlen and Newsome, 2001).

**Figure 1.**
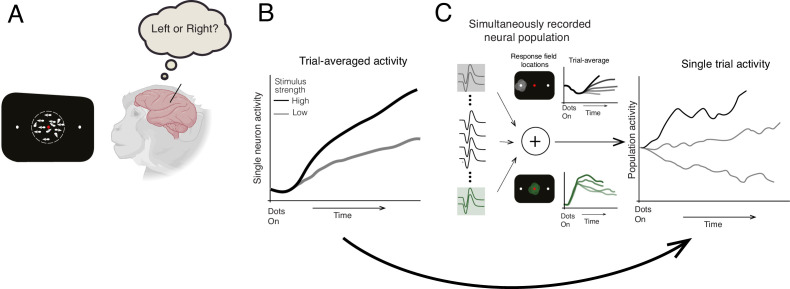
Identifying how decisions are made in the brain. (**A**) Steinemann et al. recorded data from a macaque performing a direction discrimination task where it had to decide whether a cloud of dots was moving towards a target on the left- or right-hand side of a screen (represented as a black rectangle). (**B**) Previous studies of the same task suggested that when the activity of single neurons was averaged across multiple trials, the dynamics leading to a decision followed the drift-diffusion model (ramp-like activity). When stimulus strength was high (black line), the evidence for making the decision was stronger in one direction. This caused neuronal activity to increase more rapidly and a decision to be made faster compared to when stimulus strength was low (gray line) (**C**) Steinemann et al. recorded the activity of up to 203 neurons simultaneously in the lateral intraparietal area of a macaque’s brain. The experiment identified two subpopulations of neurons that are involved in different aspects of the decision-making process. The first subgroup (gray lines, top graph) had a response field (light gray circle on black screen) that overlapped with the chosen target (small white dot) and represents the gradual accumulation of information. The second group (green lines, bottom graph) had a response field (green circle) that overlapped with the movement of the dots and represents the momentary pieces of evidence integrated into the decision. The neuronal activity of the first subgroup resembled the drift-diffusion dynamics observed in previous studies. Critically, Steinemann et al. also observed the same ramp-like dynamics when analyzing data from a single trial (right graph) providing further support for the drift-diffusion model. Note that the single-trial activity exhibits random fluctuations unlike the trial averaged activity, which is a signature of diffusion dynamics.

However, whether neurons in the LIP actually display drift-diffusion dynamics has been debated as other mechanisms can lead to similar patterns of activity when using trial-averaged data (Latimer et al., 2015; Chandrasekaran et al., 2018; Zoltowski et al., 2019). Resolving this debate requires directly observing the diffusion aspect of the signal, which is random and highly variable, and therefore averaged out when neural activity is averaged across trials. Now, in eLife, Michael Shadlen (Columbia University and Howard Hughes Medical Institute) and colleagues – including Natalie Steinemann and Gabriel Stine as joint first authors – report that simultaneously recording the activity of many neurons in a single trial confirms that drift-diffusion dynamics underlie decision-making (Steinemann et al., 2024).

Recent advances in technology have made it possible to monitor the activity of larger populations of neurons simultaneously (Jun et al., 2017). This allowed Steinemann et al. to record up to 203 neurons in the LIP area at the same time, as opposed to the individual neurons in previous studies. The firing rate of these neurons was measured while macaques performed the aforementioned direction discrimination task. In control experiments, the team identified neurons that responded before the animals moved their eyes towards an area of the visual field known as the ‘response field’. A subset of this neuronal population had a response field that overlapped with the position of the chosen target, and previous work had shown that these neurons display drift dynamics when averaged across multiple trials (Shadlen and Newsome, 2001; Figure 1B). Steinemann et al. discovered that when a sufficient number of the subset were recorded simultaneously, the same dynamics could also be observed in single trials (Figure 1C). This implies that the pattern of neuronal activity that was detected in previous studies does indeed align with the drift-diffusion model. Importantly, this behavior was not just a consequence of selecting this subset of neurons. Even when the activity of the full neuronal population was analyzed – akin to the main flight direction of the flock of birds – drift-diffusion dynamics were observed.

These findings provide strong evidence that drift-diffusion dynamics are a fundamental feature of how neuronal populations in the LIP area of the brain represent a decision. In principle, it could still be possible that individual neurons exhibit different dynamics that manifest only as drift at the population level. For instance, individual birds might move in different patterns, but collectively the flock makes a turn. But it is questionable whether such different dynamics would be behaviorally relevant given that the dynamics of the population as a whole show drift-diffusion.

Beyond the previously described subset of neurons, Steinmann et al. identified another subgroup that responded selectively to the movement direction of the patch of dots, even when the macaques did not have to make a decision about their direction. The response field of this second subset overlapped with the location of the patch of dots on the screen, suggesting that their activity represents the momentary sensory evidence that accumulates in the drift-diffusion model.

The analyses Steinmann et al. used to identify these two subsets of neurons relied on hypotheses from the drift-diffusion model and required hypothesis-driven control experiments. When the team used a more common analysis method to study neuronal populations – which was hypothesis-free and data-driven – they did not identify the first subset of neurons as being critical to the decision-making process. Interestingly, most of the recorded neurons also did not fall in to either of the two identified subgroups. This highlights the value of developing concrete hypotheses and testing the same neuronal populations in a variety of task settings. It also raises the possibility that there may be other computations in the LIP area of the brain that remain to be discovered (Freedman and Assad, 2009; Okazawa et al., 2021).

Being able to observe decision dynamics in single trials also opens the door to investigating how non-sensory components that differ between trials – such as internal states or spontaneously fluctuating biases – influence how decisions are computed in the brain. Beyond testing mathematical models, these experiments will shed light on the fundamental physiological processes underlying decision making and other cognitive functions.
